# Supplementary material for: Speed modulations in grid cell information geometry
Source: Nat Commun. 2025 Aug 19;16:7723. doi: 10.1038/s41467-025-62856-x (PMC12365221; doi:10.1038/s41467-025-62856-x)
Supplement: Supplementary file 1 — Supplementary Information [file 41467_2025_62856_MOESM1_ESM.pdf]

Supplementary Information

Supplementary Methods: Mathematical details of Bayesian Linear Ensemble Averaging

First, we consider only one sampled dataset $\mathcal{D}_s$. From this dataset, we obtain a metric-speed dataset (e.g. SCA-speed in Figure 1C), denoted as $\{t_i, \boldsymbol{x}_i\}$, where t_i is the metric value and $\boldsymbol{x_i} = (v_i, 1)$ with v_i denoting the speed. Assuming

$$t = \boldsymbol{w}^T \boldsymbol{x} + \boldsymbol{\epsilon} \tag{1}$$

where $\boldsymbol{\epsilon} \sim \mathcal{N}\left(\boldsymbol{\epsilon} \middle| 0, {\beta_{t,s}}^{-1}\right)$, $\beta_{\mathrm{t,s}}$ is a scalar representing precision. Bayesian Linear Regression (BLR) is used to obtain the posterior distribution $p(\boldsymbol{w}|\mathcal{D}_s)$, which is a Gaussian distribution $\mathcal{N}\left(\boldsymbol{w}; \boldsymbol{m}_{w,s}, \Sigma_{w,s}\right)$. Substituting this back to (1), we obtained the predictive distribution[1] $\mathrm{p}\left(t_q \middle| \boldsymbol{x}_q, \mathcal{D}_s\right)$ as $\mathcal{N}\left(t_q; m_{t,s}, \Sigma_{t,s}\right)$, where $m_{t,s} = \boldsymbol{m}_{w,s}^T \boldsymbol{x}_q$ and $\Sigma_{t,s} = \boldsymbol{x}_q^T \Sigma_{w,s} \boldsymbol{x}_q + \beta_{t,s}^{-1}$.

Next, we consider the whole dataset $\mathcal{D}$, taking into account all $\mathcal{D}_s$. Each $\mathcal{D}_s$ is a random sampling of $\mathcal{D}$, therefore, $p(\boldsymbol{w}|\mathcal{D}) = \sum_s p(\boldsymbol{w}|\mathcal{D}_s) p(\mathcal{D}_s|\mathcal{D}) = \sum_s p(\boldsymbol{w}|\mathcal{D}_s)/B$, where $B = 50$ is the number of samplings. This distribution is a mixture of the Gaussian, we approximated it as a single Gaussian function with the same mean and covariance. The mean of $p(\boldsymbol{w}|\mathcal{D})$ is

$$\boldsymbol{m}_w = \frac{1}{B} \sum_s \boldsymbol{m}_{w,s} \tag{2}$$

The covariance is

$$\begin{aligned} \Sigma_w &= \int p(\boldsymbol{w}|\mathcal{D})(\boldsymbol{w} - \boldsymbol{m}_w)(\boldsymbol{w} - \boldsymbol{m}_w)^T d\boldsymbol{w} \\ &= \frac{1}{B} \sum_s \int p(\boldsymbol{w}|\mathcal{D}_s)\left(\boldsymbol{w} - \boldsymbol{m}_{w,s} - \boldsymbol{m}_w\right)\left(\boldsymbol{w} - \boldsymbol{m}_{w,s} - \boldsymbol{m}_w\right)^T d\boldsymbol{w} \\ &= \frac{1}{B} \sum_s \Sigma_{w,s} + \frac{1}{B} \sum_s \left(\boldsymbol{m}_{w,s} - \boldsymbol{m}_w\right)\left(\boldsymbol{m}_{w,s} - \boldsymbol{m}_w\right)^T \end{aligned} \tag{3}$$

We can use the same trick to compute the mean and covariance of the predictive distribution. The mean is

$$m_t = \frac{1}{B} \sum_s m_{t,s} = \left(\frac{1}{B} \sum_s \boldsymbol{m}_{w,s}^T\right) \boldsymbol{x}_q = \boldsymbol{m}_w^T \boldsymbol{x}_q \tag{4}$$

The covariance is

$$\begin{aligned} \Sigma_t &= \frac{1}{B} \sum_s \Sigma_{t,s} + \frac{1}{B} \sum_s \left(m_{t,s} - m_t\right)^2 \\ &= \frac{1}{B} \sum_s \Sigma_{t,s} + \frac{1}{B} \sum_s \boldsymbol{x}_q^T \left(\boldsymbol{m}_{w,s} - \boldsymbol{m}_w\right)\left(\boldsymbol{m}_{w,s} - \boldsymbol{m}_w\right)^T \boldsymbol{x}_q \\ &= \boldsymbol{x}_q^T \Sigma_w \boldsymbol{x}_q + \beta_t^{-1} \end{aligned} \tag{5}$$

where $\beta_t^{-1} = \sum_s \beta_{t,s}^{-1} / B$. Inspecting the mean and covariance of the predictive distribution, it is clear that even considering the whole dataset $\mathcal{D}$, metric is still a linear function of speed, written explicitly as

$$t_q = \boldsymbol{w}^T \boldsymbol{x}_q + \epsilon \qquad (6)$$

where $\boldsymbol{w} \sim \mathcal{N}(\boldsymbol{w}; \boldsymbol{m}_w, \Sigma_w)$ and $\epsilon \sim \mathcal{N}\left(\epsilon; 0, \sum_s \beta_{t,s}^{-1} / B\right)$

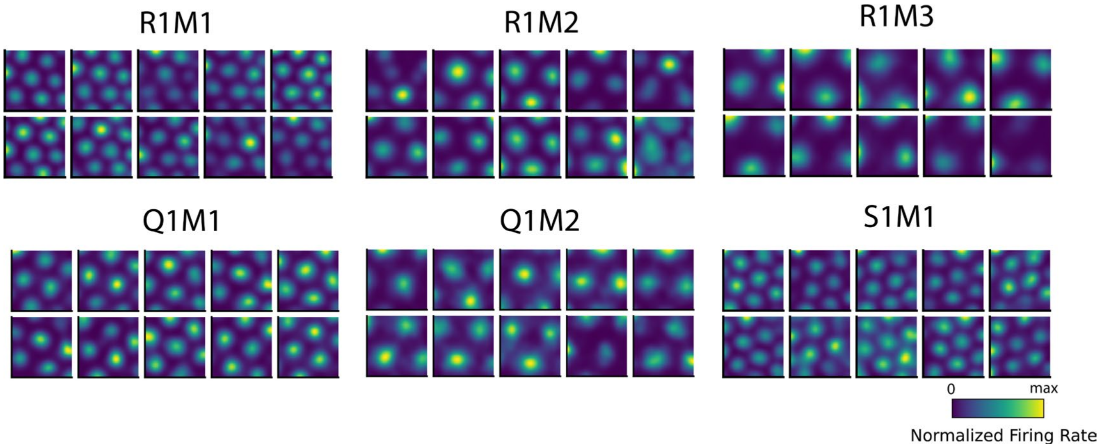

Supplementary Figure 1. Example grid cells' rate maps from different datasets (see Methods).

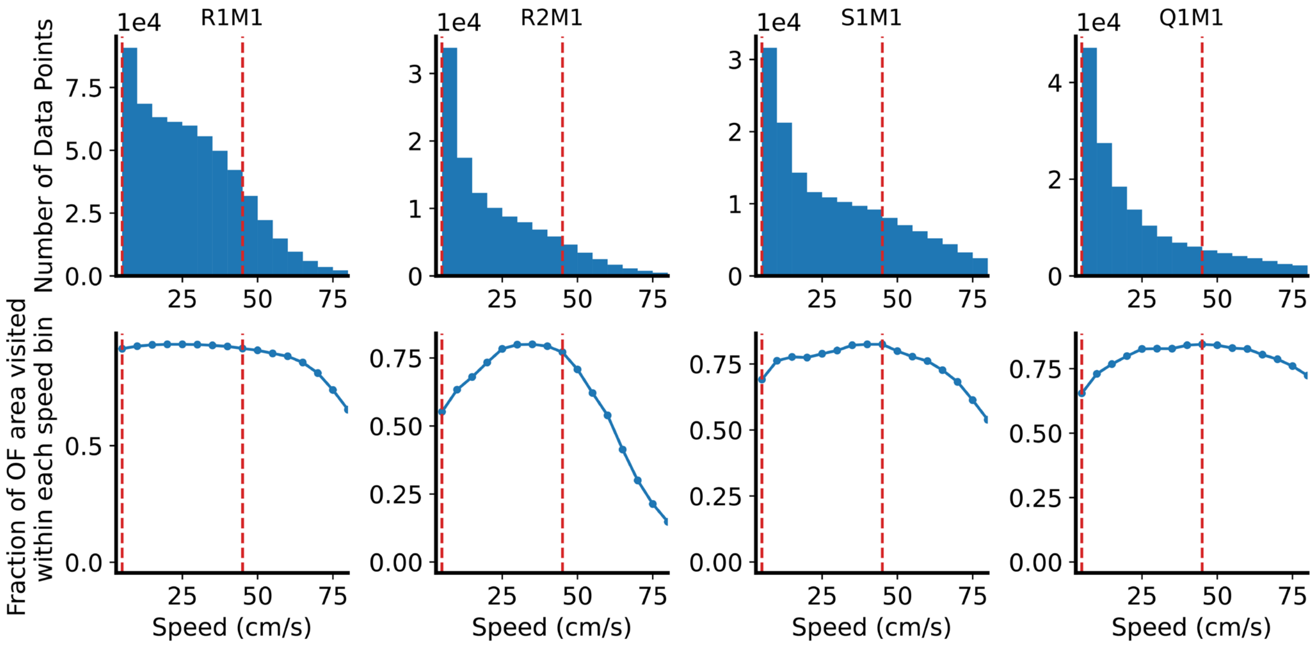

Supplementary Figure 2. Statistics of behavior labels (x, y locations and speed). Upper: Number of data points in each small speed bin (bin width = 5 cm/s). Each data point represents a neural state at a 10 ms time bin. Two vertical dashed lines enclose the speed range considered in this paper (5 cm/s to 45 cm/s). The statistics for R1M2 and R1M3 are the same as R1M1; R2M2 and R2M3 are the same as R2M1; Q1M2 is the same as Q1M1. Bottom: The entire OF area is digitized into 30-by-30 spatial bins. The y-axis indicates the fraction of bins visited by the rat within a speed bin. Source data are provided as a Source Data file.

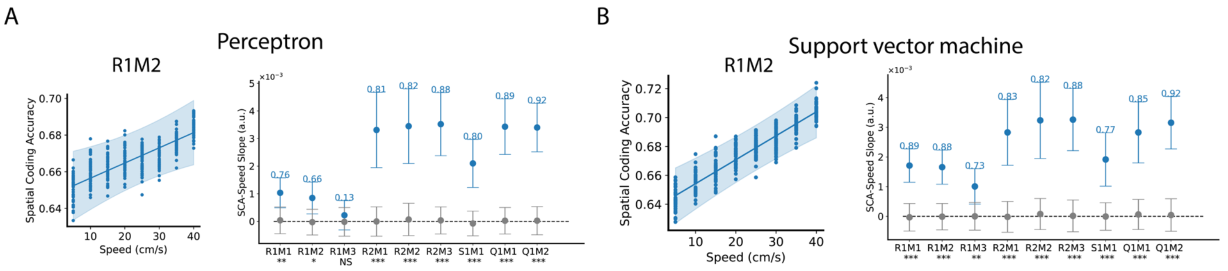

Supplementary Figure 3. Grid cell population spatial coding accuracy (SCA) improves with increasing speed. (**A, B**) Same as Figure 1C, 1D, but using perceptron (panel A) and support vector machine (panel B, L2 regularization with coefficient C=1, implemented by the sklearn.svm.svc class) for computing the SCA (see Figure 1B). Fifty sample data sets $\mathcal{D}_s$ are used. Source data are provided as a Source Data file.

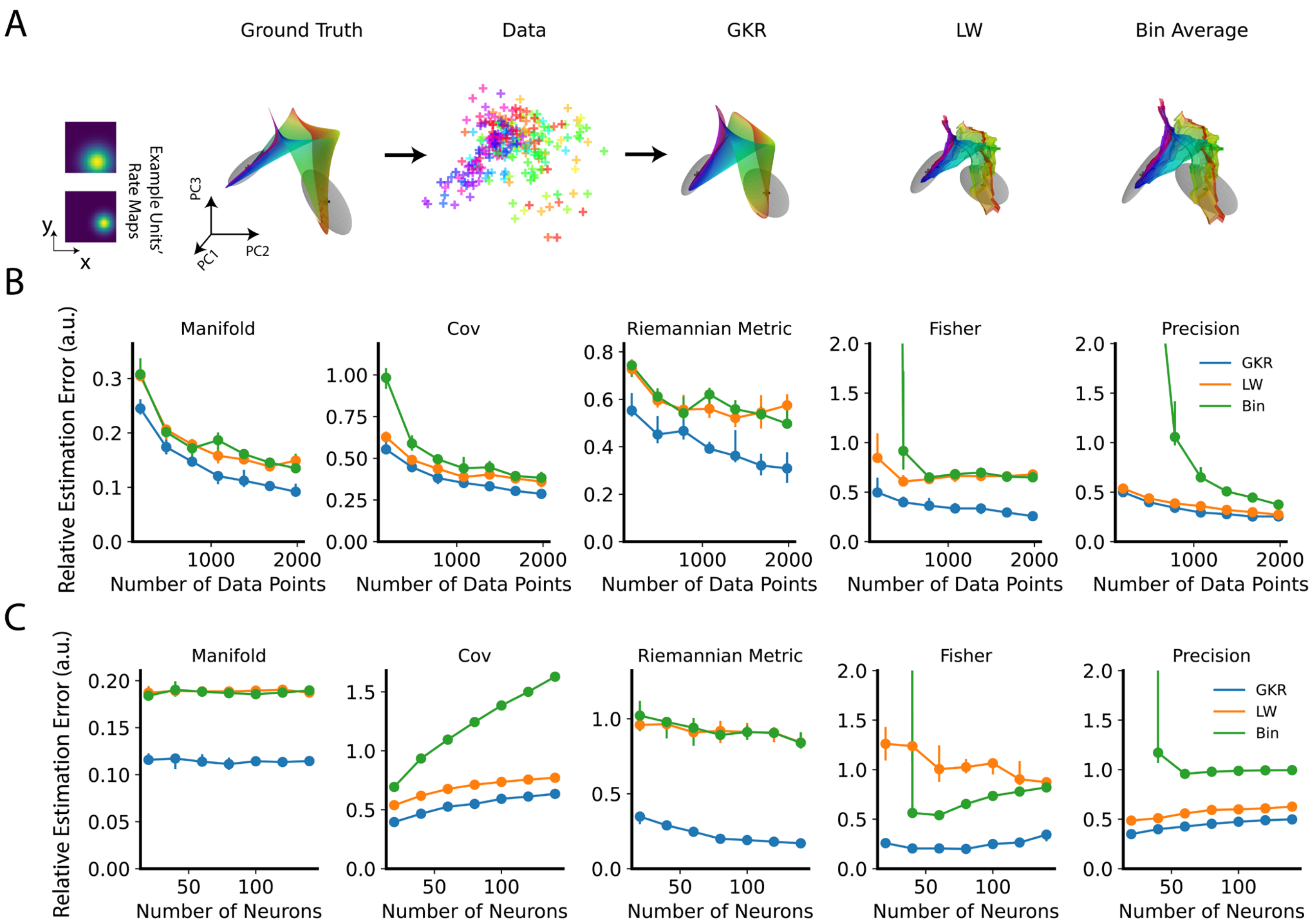

Supplementary Figure 4: Testing GKR on a 2D synthetic manifold. (**A**) The synthetic dataset comprises N synthetic neurons with heterogeneous tuning maps on a 2D space $\boldsymbol{p}$. Ground truth $\boldsymbol{\mu}(\boldsymbol{p})$ and $\Sigma(\boldsymbol{p})$ were visualized using the first three principal components, shown on the left. Ellipsoid axes represent the directions of three covariance eigenvectors, with lengths proportional to the eigenvalues. In this example, the synthetic dataset has 10 neurons and generated 200 data points. These data points were then fed into different methods for fitting manifold. (**B, C**) Evaluation of different methods' performances under various conditions. The default number of data points is 1,000, and the default number of neurons is 10. The illustration of these panels is the same as in main Figure 2C, D. Source data are provided as a Source Data file.

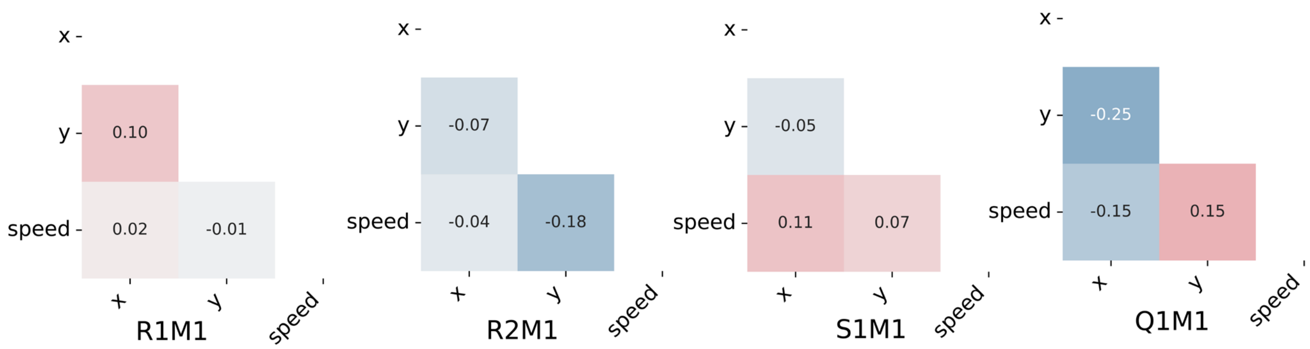

Supplementary Figure 5. Correlation of behavior labels. We computed the Pearson correlation for each pair of behavior labels during a recording session. Note that grid cells from different modules but within the same recording session share identical behavior label values, for example, datasets R1M1 and R1M2 have identical behavior label values.

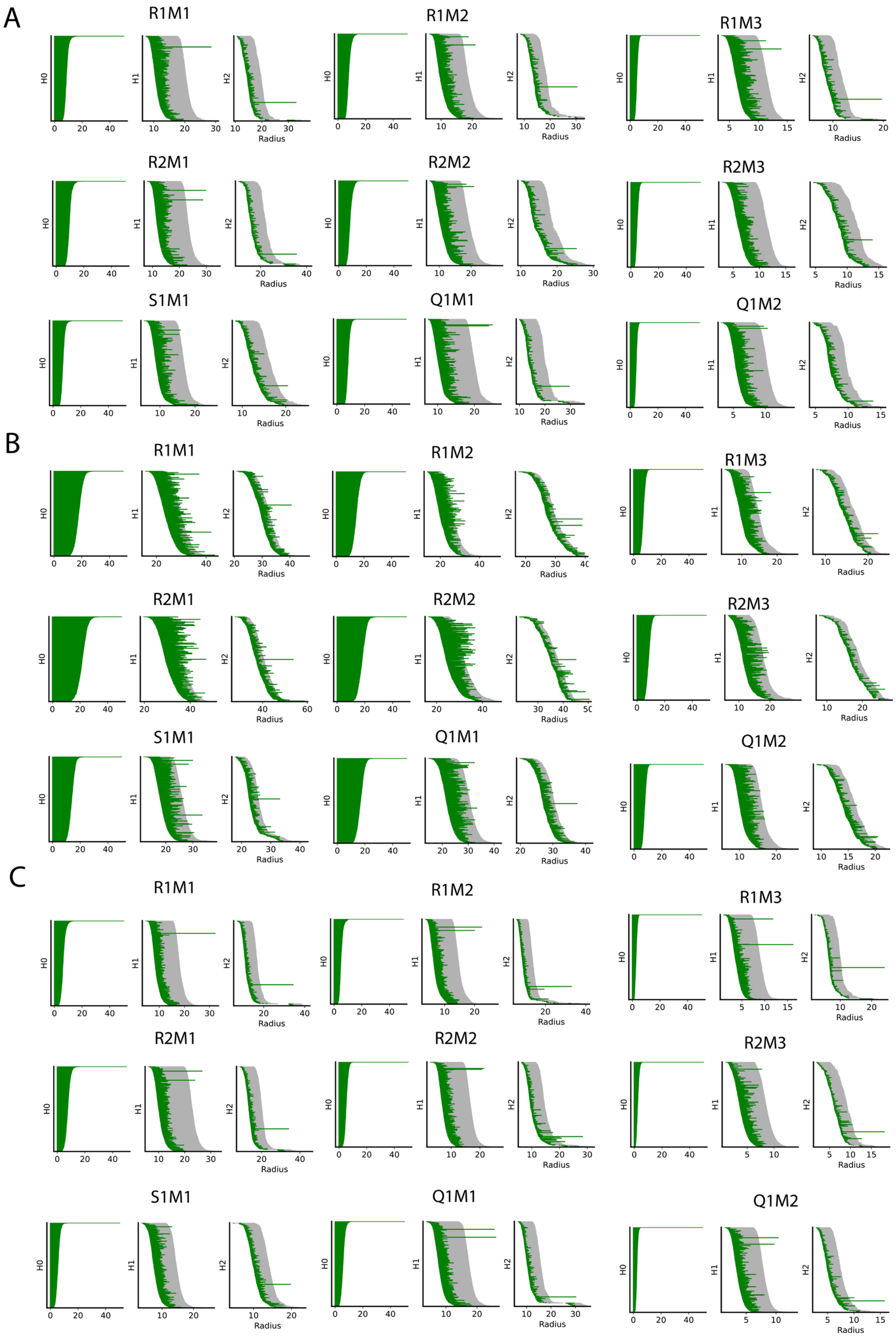
A
B
C
R1M1
R1M2
R1M3
R2M1
R2M2
R2M3
S1M1
Q1M1
Q1M2
H0
H1
H2
Radius

Supplementary Figure 6. Grid cell population forms toroidal-like manifold. (**A**) Persistent homology barcode for topological analysis. Long bars represent possible true topological structures. H0, H1, and H2 indicate a connected component, a circular hole, and a cavity, respectively. A torus is characterized by one long bar in H0, two in H1, and one in H2. The sampled dataset $\mathcal{D}_S$ was used to fit a GKR model. The fitted manifold is intrinsically three-dimensional (with three labels: x location, y location, and speed). We randomly sampled 6,400 label points and input them into the GKR model to predict 6,400 manifold points in the original high-dimensional space (where the number of dimensions equals the number of grid cells). These manifold points were then reduced to their first six PC dimensions. These dimensionally reduced manifold points were clustered into 1,200 centers using k-means clustering. These 1,200 cluster centers were then analyzed using persistent homology, as shown by the barcode in the figure. Grey bars indicate the maximum bar lengths from 20 shuffles of the 1,200 cluster centers (see Methods). (**B**) Same as (A), but without PCA dimension reduction. (**C**) Similar to (A), but with speed fixed at 20 cm/s. At this speed, 30-by-30 grid points were sampled in the OF field, fed into GKR to predict 900 manifold points, which were then projected onto the first six PC dimensions and analyzed using persistent homology (see Methods). Grey bars indicate the maximum bar lengths from 20 shuffles of the 900 manifold points (see Methods).

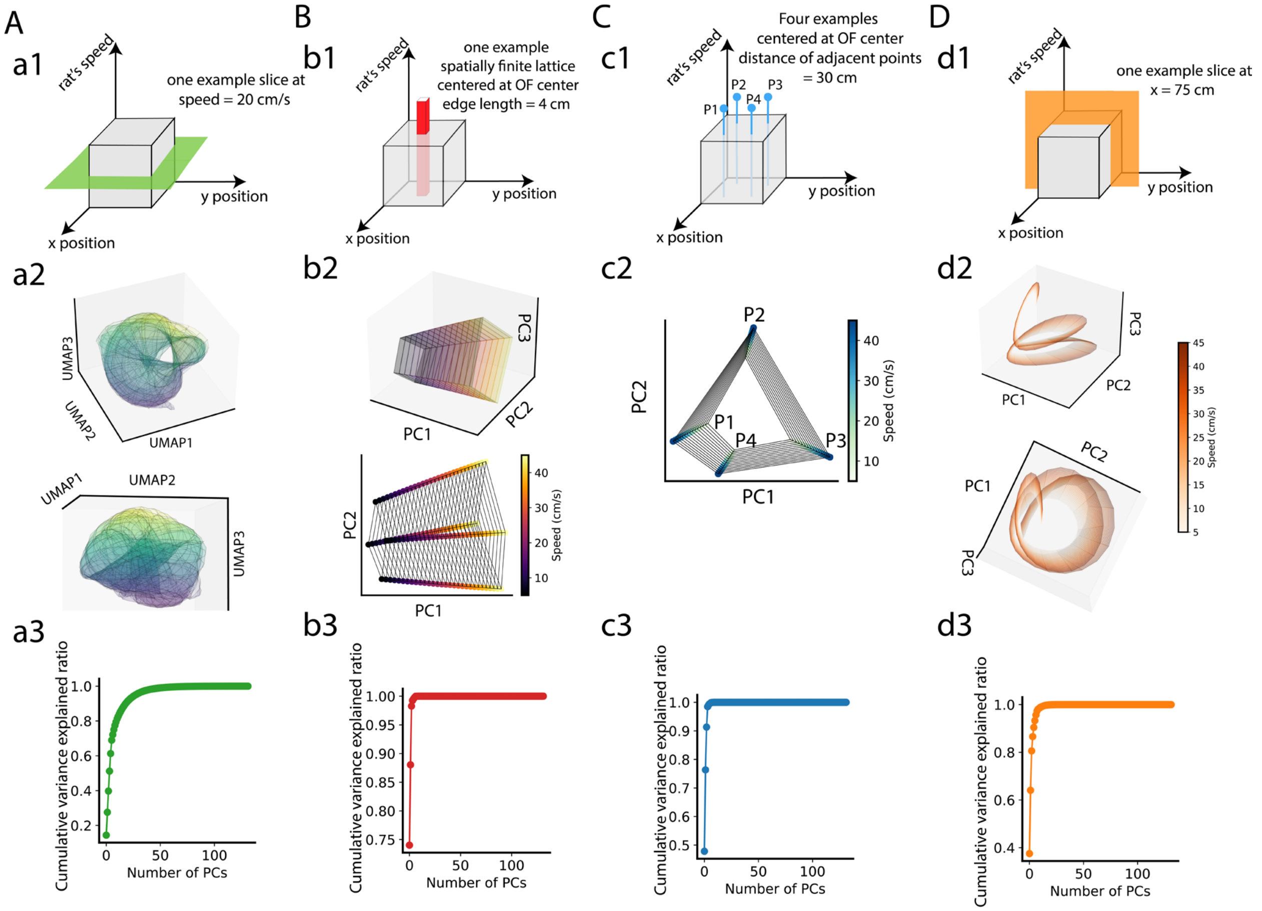

Supplementary Figure 7. Visualization of different manifold slices of a GKR fitted from R1M2. (**A**) a1: The speed slice; a2: Two views of the manifold, visualized by first projecting the manifold to the 6 PC space, then non-linearly reduced to three dimensions using UMAP; a3: Cumulative variance explained ratio of the manifold. (**B**) b1: Example lattice centered at the OF center with edge lengths = 4 cm; b2: Manifold visualization by projecting the manifold onto the first three (and two) principal components; b3: Cumulative variance explained ratio of the manifold. (**C**) c1: Four example spatial points; c2: Manifold visualization by projecting the manifold onto the first two principal components; c3: Cumulative variance explained ratio. (**D**) d1: Fixing x position value while varying y position and running speed; d2: Two views of the manifold, vitalized by projecting the manifold to the first three principal components; d3: Cumulative variance explained ratio.

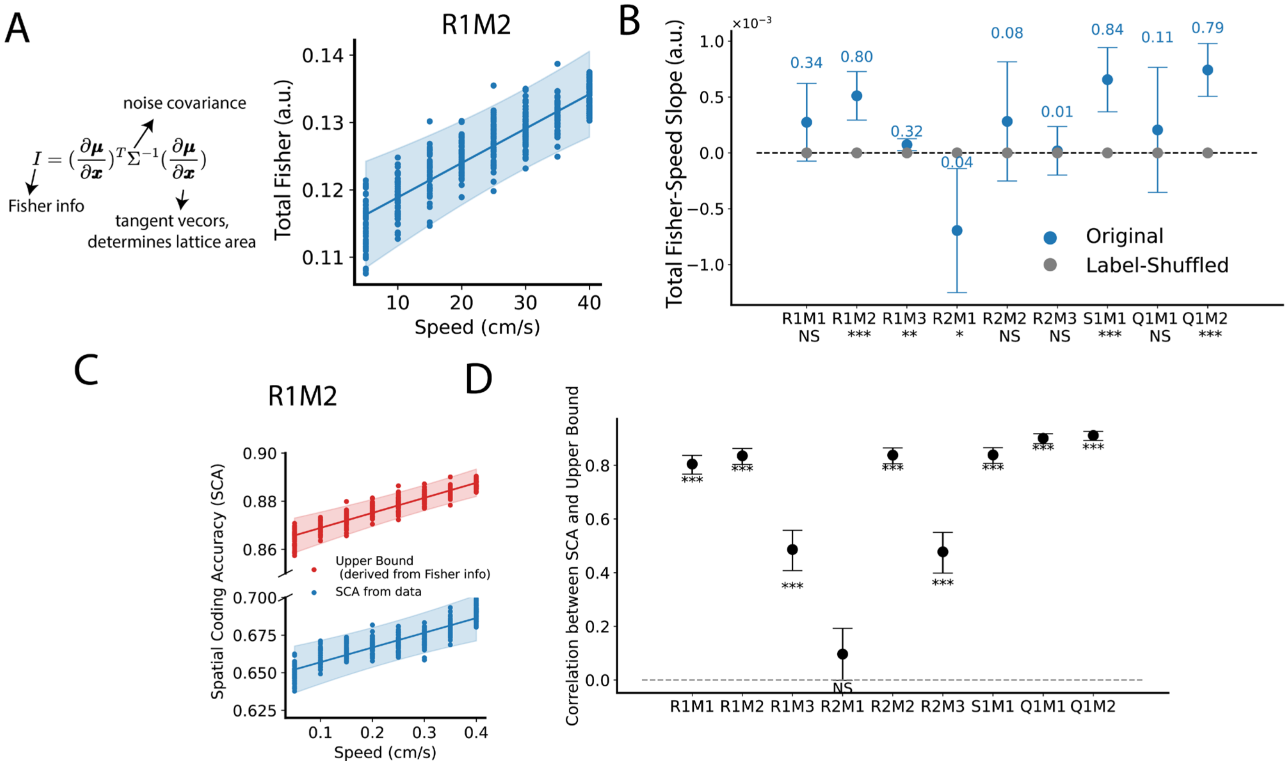

Supplementary Figure 8. Speed modulation of Fisher information computed from the original high-dimensional space (dimensionality equal to the number of grid cells). (**A, B, C, D**) Same illustrations as Figure 5A, B, C, D, respectively, but using the original $\mathcal{D}_s$ without PCA reduction. Source data are provided as a Source Data file.

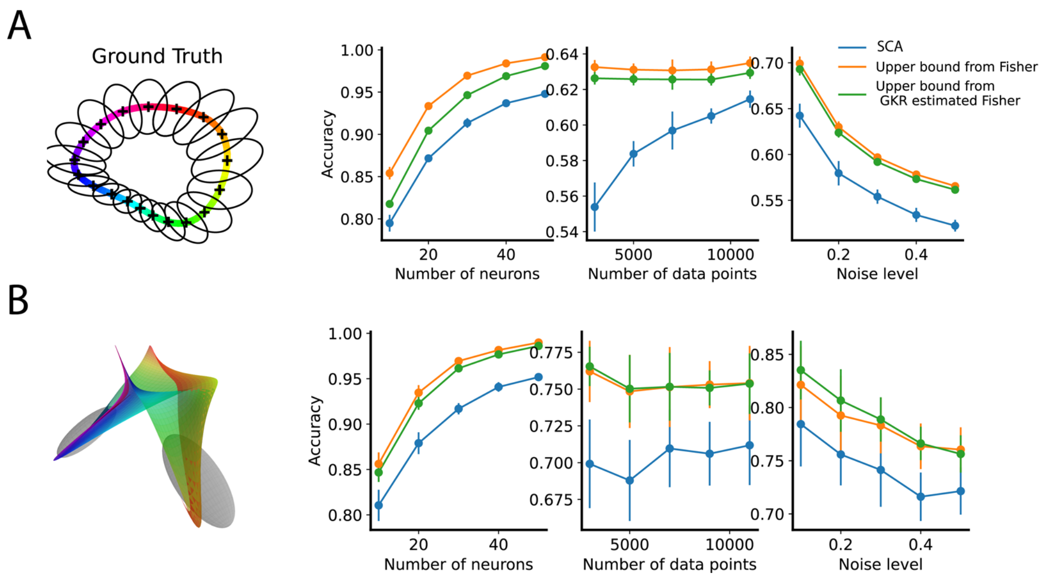

Supplementary Figure 9. Testing upper bounds of the SCA derived from Fisher information on synthetic datasets. (**A**) The default parameters are 5 neurons, 5000 data points, and noise level ν = 0.2. Other parameters are detailed in Methods. For each condition, data points were input into GKR, which output estimated Fisher information. The estimated Fisher information was then used to compute the SCA upper bound (see Methods). We also computed the upper bound using ground truth Fisher and directly calculated SCA from the raw data points (see Methods). Dots and error bars represent the median, first, and third quantiles from 10 randomly generated synthetic datasets. (**B**) Same as (A) but using 2D synthetic datasets. The default parameters are 5 neurons, 10,000 data points, and noise level ν = 0.5. Other parameters are detailed in Methods. Source data are provided as a Source Data file.

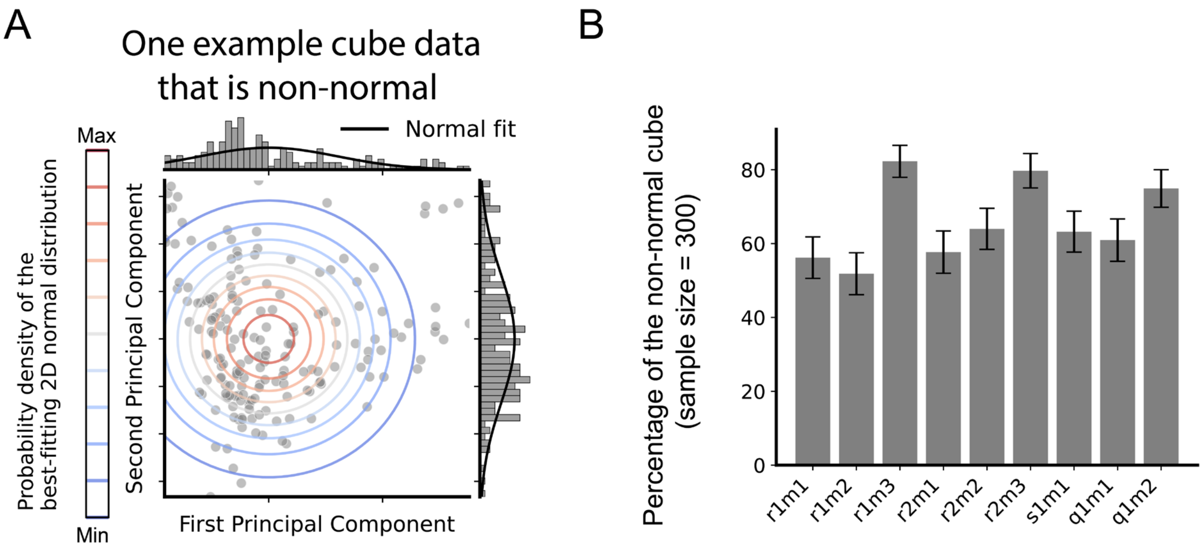

Supplementary Figure 10. Assessing the normality of the data. (**A**) Visualization of an example cube of data. A cube was randomly sampled in the label space with edge lengths of (10 cm, 10 cm, 10 cm/s). Data within the cube, obtained from R1M2, were collected and projected onto PC1 and PC2 axes. The central panel shows the 2D projection, while the histograms on the top and right display the marginal distributions along the two PC axes. Black lines in the side panels and the contours in the central panel represent the optimal normal distribution fitted via maximum likelihood estimations. A Henze–Zirkler test applied to the 2D data and Shapiro-Wilk tests applied to the two 1D projections indicate that this sampled cube does not follow a normal distribution (which we require the p-values for all tests < 0.05). This cube is also referred to as a "non-normal cube." (**B**) To systematically assess normality across experimental configurations (e.g., R1M2 is a configuration), we randomly sampled 300 cubes per configuration. For each sampled cube, data were projected onto a randomly selected PC, and processed by a Shapiro-Wilk test to evaluate its normality. Bar plot shows the percentages of non-normal cubes. Error bars indicate the 95% confidence intervals (Wald interval). Source data are provided as a Source Data file.

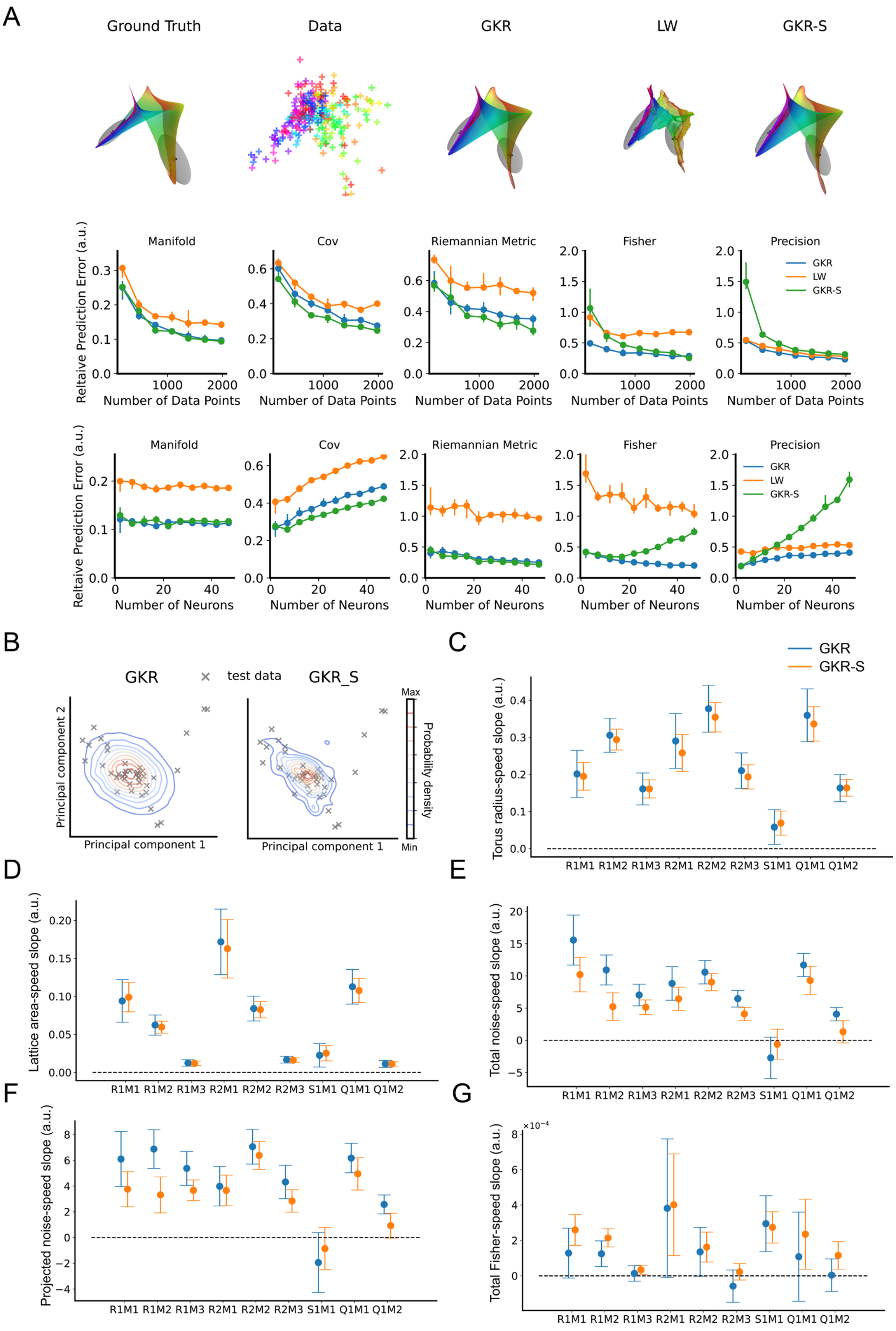
A
Ground Truth
Data
GKR
LW
GKR-S
Manifold
Cov
Riemannian Metric
Fisher
Precision
Reltaive Prediction Error (a.u.)
Number of Data Points
Number of Neurons
GKR
LW
GKR-S
B
GKR
test data
GKR_S
Principal component 1
Principal component 2
Max
Min
Probability density
C
GKR
GKR-S
Torus radius-speed slope (a.u.)
R1M1 R1M2 R1M3 R2M1 R2M2 R2M3 S1M1 Q1M1 Q1M2
D
Lattice area-speed slope (a.u.)
E
Total noise-speed slope (a.u.)
F
Projected noise-speed slope (a.u.)
G
Total Fisher-speed slope (a.u.)
×10⁻⁴

Supplementary Figure 11. Reproducing key results using GKR-S which does not assume data normality. (**A**) GKR-S is a method similar to GKR but does not assume that the noise distribution follows a normal distribution. The first step of GKR-S is the same as GKR, using Gaussian process regression. The second step of GKR employs a non-parametric resampling approach. To estimate the conditional distribution at a single label point, it resamples data points from adjacent labels with resampling prior assigned by a kernel. The resulting resampled data points can then be used to estimate distribution statistics (see *Methods: Gaussian process regression with kernel sampling (GKR-S)*). Here, we evaluated GKR-S on a 2D synthetic dataset using the same illustration style and parameter configurations as Supplementary Figure 4. We did not show the results of the bin-average method as which often fall outside the panel. Although we use ellipsoids to represent noise covariances, GKR-S does not assume the noise to be normal. (**B**) Example illustrations of the marginal distributions fitted by GKR and GKR-S from a grid cell dataset. Since GKR-S performs well only in low-dimensional spaces (see panel A), instead of fitting the full dimensional dataset $\mathcal{D}_s$ (from R1M2), we projected $\mathcal{D}_s$ onto its first six PCs, denoted as $\mathcal{D}_s^{(6)}$ (the choice of six PCs is align with a prior work[2]). $\mathcal{D}_s^{(6)}$ was split evenly into training and test sets. The training set was used to fit the conditional distribution $p(\boldsymbol{r}|\boldsymbol{x})$ via GKR or GKR-S. To illustrate the "shape" of the fitted distribution density function, we randomly selected an example cube centered at $\boldsymbol{x}_{eg} = (84 \text{ cm}, 45 \text{ cm}, 23 \text{ cm/s})$ with edge lengths of (10 cm, 10 cm, 10 cm/s). Test data within this cube were collected and projected onto their first two PCs for visualization (crosses). On the other hand, we generated 10000 sampling points from the distribution $p(\boldsymbol{r}|\boldsymbol{x}_{eg})$, and projected them onto the same PC1-PC2 plane. The projected samples were smoothed using a kernel density estimation, providing an estimated marginal distribution on the PC1-PC2 plane, visualized as contours in this panel. (**C–G**) We conducted the same analysis as in Figures 3, 4, and 5 to recompute the key results in this study. The illustrations follow the same format as Figures 3, 4, and 5. The results from GKR-S are quantitatively consistent with those obtained using the GKR method. Source data are provided as a Source Data file.

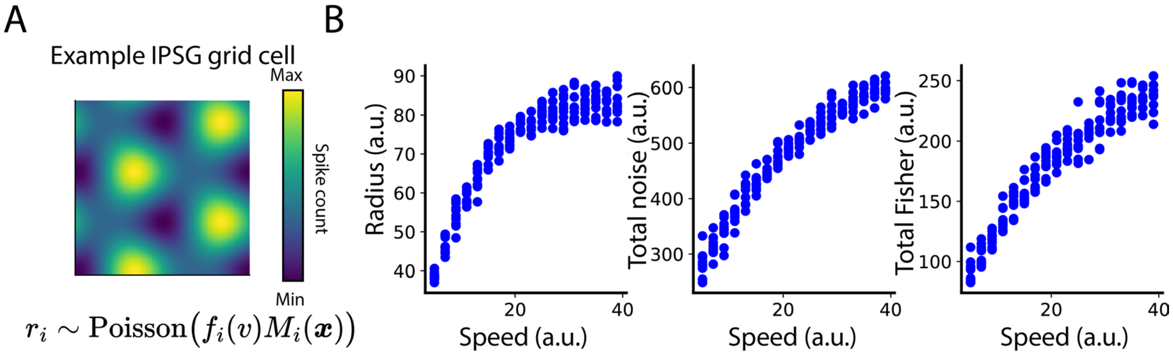

Supplementary Figure 12. The effects of speed modulation on grid cell information geometry can be qualitatively reproduced using an Independent Poisson Speed-Gain (IPSG) model. (**A**) The IPSG model assumes that grid cells fire independently, with their rate maps modulated by running speed via a monotonically increasing gain factor (see Methods: *Simulation of IPSG grid cells*). r_i: spike counts; $f_i(v)$: gain factor; $M_i(x)$: spatial rate map, where index i indicate neuron i. This panel shows an example rate map of an IPSG grid cell at a speed of 10. (**B**) We simulated spike trains from a population of IPSG grid cells (n=10), generating a dataset of spike counts with corresponding position and speed. To estimate uncertainty, we performed 10 resampling with replacement. Each resampled dataset was used to fit a GKR model, which was then used to estimate the manifold radius, total noise, and total Fisher information. Each dot represents the result from one resampled dataset at a given speed. Source data are provided as a Source Data file.

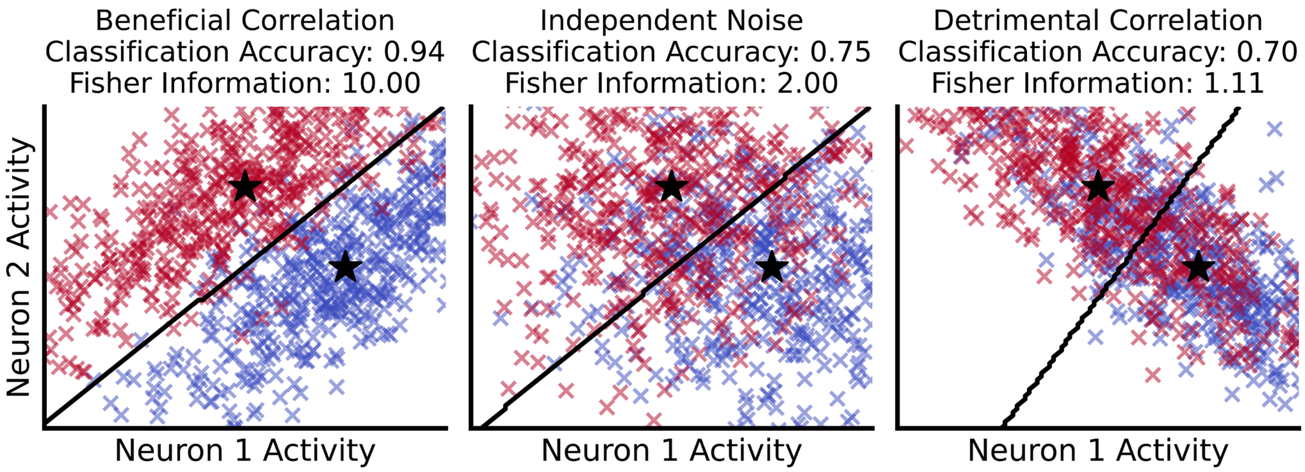

Supplementary Figure 13. A two-neuron system illustrating how the effects of noise correlation depend on the geometry of noise covariance. We constructed a simple two-neuron system to encode two classes, with firing rates following a 2D Gaussian distribution (see *Methods: A two-neuron example demonstrating the effects of noise correlation*). The mean firing rates for each class are represented by the two stars. To examine the impact of noise correlations, we constructed three covariance matrices with distinct correlation coefficients (from left to right), and in each condition the total noise variance was held constant at 2. For each condition, we sampled 1,000 data points (500 per class, part of the data points are indicated by crosses), and trained a logistic regression classifier to distinguish the two classes. The results demonstrate that noise correlation can either enhance (left) or impair (right) information encoding, depending on the geometry of the noise covariance matrix. These findings can be interpreted in analogy to Figure 6. In real neural populations, noise correlation is present (as in the left or right case). If we manually remove noise correlation by setting the off-diagonal terms of the noise covariance matrix to zero, the system transitions to the middle case. This manipulation can either increase Fisher information (right) or decrease it (left), depending on the original correlation structure. In Figure 6, we show that the grid cell population more closely resembles the right case, where removing noise correlation enhances Fisher information. Source data are provided as a Source Data file.

SI References

1. Bishop, C. M. *Pattern Recognition and Machine Learning*. (Springer New York, 2006).

2. Gardner, R. J. *et al.* Toroidal topology of population activity in grid cells. *Nature* **602**, 123–128 (2022)

   DOI: 10.1038/s41586-021-04268-7.
